# Involvement of Cytokines and Hormones in the Development of Spermatogenesis In Vitro from Spermatogonial Cells of Cyclophosphamide-Treated Immature Mice

**DOI:** 10.3390/ijms22041672

**Published:** 2021-02-07

**Authors:** Ronnie Solomon, Ali AbuMadighem, Joseph Kapelushnik, Bat-Chen Amano, Eitan Lunenfeld, Mahmoud Huleihel

**Affiliations:** 1 The Shraga Segal Department of Microbiology, Immunology, and Genetics, Faculty of Health Sciences, Ben-Gurion University of the Negev, Beer Sheva 8410501, Israel; ronsol2410@gmail.com (R.S.); abumadig@post.bgu.ac.il (A.A.); amanub@post.bgu.ac.il (B.-C.A.); 2 The Center of Advanced Research and Education in Reproduction (CARER), Faculty of Health Sciences, Ben-Gurion University of the Negev, Beer Sheva 8410501, Israel; kapelush@bgu.ac.il (J.K.); lunenfld@bgu.ac.il (E.L.); 3 Department of Pediatric Oncology and Department of Hematology, Soroka Medical Center, Beer Sheva 8410501, Israel; 4 Department of OB/GYN, Soroka Medical Center, Beer Sheva 8410501, Israel

**Keywords:** spermatogenesis, male infertility, cyclophosphamide, in vitro maturation of spermatogonial cells, chemotherapy, cytokines, hormones

## Abstract

Aggressive chemotherapy treatment may lead to male infertility. Prepubertal boys do not produce sperm at this age, however, they have spermatogonial stem cells in their testes. Here, we examined the effect of intraperitoneal injection of cyclophosphamide (CP) on the capacity of immature mice (IM) to develop spermatogenesis in vivo and in vitro [using methylcellulose culture system (MCS)]. Our results show a significant decrease in testicular weight, total number of testicular cells, and the number of Sertoli, peritubular, premeiotic, and meiotic/post-meiotic cells, but an increase in the percentages of damaged seminiferous tubules in CP-treated IM compared to control. The functionality of Sertoli cells was significantly affected. The addition of testosterone to isolated cells from seminiferous tubules of CP-treated IM significantly increased the percentages of premeiotic (CD9-positive cells) and meiotic/post-meiotic cells (ACROSIN-positive cells) developed in MCS compared to control. The addition of FSH did not affect developed cells in MCS compared to control, but in combination with testosterone, it significantly decreased the percentages of CD9-positive cells and ACROSIN-positive cells. The addition of IL-1 did not affect developed cells in MCS compared to control, but in combination with testosterone, it significantly increased the percentages of VASA-positive cells and BOULE-positive cells compared to IL-1 or testosterone. Addition of TNF significantly increased only CD9-positive cells in MCS compared to control, but in combination with testosterone, it significantly decreased ACROSIN-positive cells compared to testosterone. Our results show a significant impairment of spermatogenesis in the testes of CP-treated IM, and that spermatogonial cells from these mice proliferate and differentiate to meiotic/post-meiotic cells under in vitro culture conditions.

## 1. Introduction

Spermatogenesis is a cyclical process which occurs within the seminiferous tubules. Spermatogonial stem cells (SSCs) are located close to the basement membrane of the seminiferous tubule; they proliferate, differentiate, and undergo meiosis and spermiogenesis to generate mature sperm [1,2,3]. Spermatogenesis is controlled by both endocrine and paracrine/autocrine systems. Luteinizing hormone (LH), follicle stimulating hormone (FSH) and testosterone are a part of the hypothalamus–pituitary–testes axis, serve as endocrine factors that control the development of complete spermatogenesis [2,3,4,5,6]. FSH prevents spermatogonial cells apoptosis, is involved in DNA synthesis, and induces germ cells differentiation in vitro [5,6]. Testosterone is involved in sex organ development and induces differentiation of spermatogonial cells [7]. Testicular autocrine/paracrine factors (growth factors/cytokines and others) are involved in the control of spermatogenesis development [1,2,3,8,9,10,11]. Interleukin-1 alpha (IL-1α) and tumor necrosis factor alpha (TNF-α) have a key role in spermatogenesis. IL-1α, secreted by Sertoli, Leydig, and germ cells [3,9,10,11,12], induces proliferation, differentiation, and survival of spermatogonial cells [13,14,15]. Several cytokines are secreted by testicular interstitial macrophages that are involved in regulation of Leydig cell steroidogenesis [10,11,16,17]. It has been shown that TNF-α inhibits Leydig cell steroidogenic acute regulatory (StAR) protein expression and steroidogenesis in a rat model with chronic systemic inflammation and sepsis [18]. TNF-α also decreases StAR protein expression and testosterone synthesis in both vehicle-treated and human chorionic gonadotropin (hCG)-treated rats [19]. Administration of TNF-α to healthy men and rodents induced a significant decrease in serum testosterone levels [20,21]. TNF-α inhibits gene expression of steroidogenic enzymes at the transcriptional level. TNF-α is produced by interstitial macrophages, round and elongating spermatids, and pachytene spermatocytes and Sertoli cells [22,23]. TNF-α binds to either one of its two receptors: TNFRI (p55, CD120a) and TNFRII (p75, CD120b), which are mostly restricted to Sertoli and Leydig cells in the testis [22,24,25,26,27]. TNF-α induces cell death of testicular germ cells (apoptosis) through its binding to TNFRI [28]. TNF-α, secreted by Sertoli and germ cells, is involved in lactate metabolism, which is the source of energy for spermatogonial cells [25,29]. It also increased the transferrin production levels by Sertoli cells, which enable germ cells to consume more iron, which is important for spermatogenesis [29]. TNF-α induces secretion of different cytokines in the testis and involved in tight junction functionality [29]. Cyclophosphamide (CP) is an alkylating agent from the nitrogen mustard family. It is commonly used for treatment of various cancers, autoimmune disorders, and graft rejection [30]. CP has diverse side effects on male fertility. It was found that exposure of mouse fetus to CP reduced their testes weights, induced testicular cancer, caused seminiferous tubules atrophy, and impaired their spermatogenesis [31]. Treatment of mouse and human with CP lead to impairment of spermatogenesis, including the development of oligospermia and even long term or permanent azoospermia [32,33]. It also may affect sperm DNA structure and may increase spontaneous miscarriage [34]. An in vitro study revealed that CP inhibited telomerase activity in mice spermatogonial germ cells [35] and led to histological alternation of the seminiferous tubules [36]. Fertility preservation of adult cancer patients is possible by cryopreservation of their sperm before chemotherapy/radiotherapy treatment. However, this is not an option for prepubertal male patients because they do not produce sperm at this age [37,38,39,40,41]. On the other hand, their testicular tissue contains SSCs that could be used for development of sperm using technologies already successful in animal models, such as testicular germ cell or organ transplantation [37,38,39,40,41,42,43]. The limitation of these technologies in cancer patients is the possibility of presence of residual cancer cells in their testicular biopsy which may lead to cancer relapse to the cure patients. If successful, in vitro differentiation of SSCs to sperm is one of the safe options for fertility preservation of prepubertal cancer patient males [the generated sperm could be used to fertilize oocyte by intracytoplasmic sperm injection (ICSI) technology]. Our group could induce the development of spermatogonial cells from normal and busulfan-treated immature mice to complete spermatogenesis including the generation of sperm-like cells using three-dimension (3D) in vitro culture systems [44,45,46,47]. Moreover, we were able to induce the development of testicular germ cells from prepubertal monkeys to round spermatid in a 3D methylcellulose culture system (MCS) [2]. Recently, we could induce the development of spermatogonial cells isolated from testicular biopsies of prepubertal cancer patient males to different stages of spermatogenesis including sperm-like cells in vitro using 3D MCS [48].

In the present study, we aimed to examine the effect of CP on the development of spermatogenesis in immature mice and its effect on the numbers of subpopulations of spermatogonial cells and Sertoli and peritubular cells (somatic cells), and also on the functionality of Sertoli cells. An additional goal was to evaluate the capacity of spermatogonial cells from CP-treated immature mice to develop spermatogenesis in vitro using 3D MCS. Here, we showed that CP treatment of immature mice impaired their spermatogenesis and significantly decreased the number of subpopulations of spermatogonial cells and Sertoli and peritubular cells, and also to affect the functionality of Sertoli cells. In addition, we were able, for the first time, to induce the proliferation of spermatogonial cells from the CP-treated immature mice and to increase their differentiation to meiotic and post-meiotic stages in vitro in the presence of cytokines and hormones in 3D MCS.

## 2. Results

### 2.1. Cyclophosphamide Significantly Decreased the Testicular Weight and Seminiferous Tubule Normal Histology and VASA, GFR-α-1, a-6-Integrin, CD9, and C-KIT Cells Counts in the Tubules of Immature Mice

**Figure 1:**
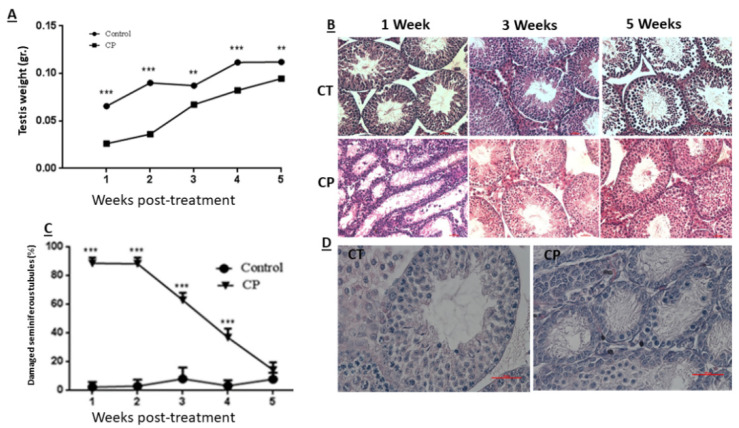

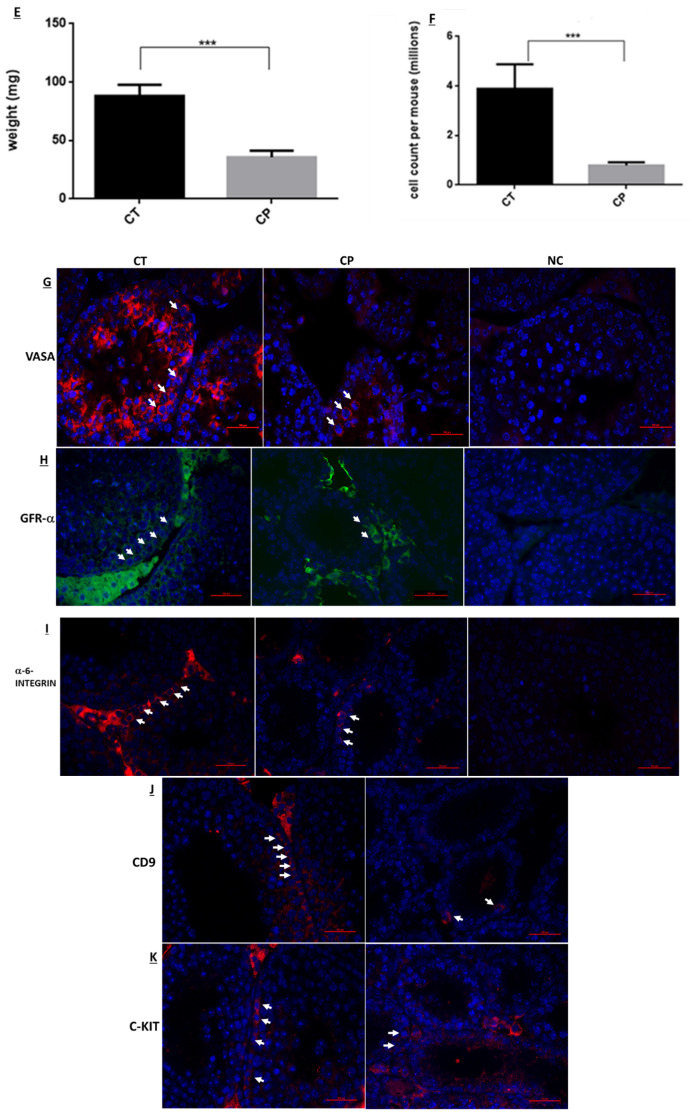
Cyclophosphamide significantly decreased the testicular weight and seminiferous tubule normal histology, VASA cells GFR-α-1, α-6-Integrin, CD9, and C-KIT cells counts in the tubules of immature mice: cyclophosphamide (CP) was intraperitoneally injected (i.p; 100 mg/kg in 100 uL; see methodology section) (CP) or PBS (control, CT; 100 uL). One to 5 weeks after the last injection, mice were sacrificed, and testes were removed, weighed, and fixed in Bouin’s solution for histological evaluation. Changes in the testes weight following CP treatment (CP) compared to control (Control) is presented (**A**). The histology of the seminiferous tubules was examined by hematoxylin-eosin staining (**B**) and a summary of seminiferous tubule damage after 1–5 weeks post CP (CP) treatment compared to the CT is presented (**C**). Ten days post-treatment, the histology of the seminiferous tubules was evaluated by H&E staining (**D**), testes were weighed (**E**), and the total number of cells isolated from the seminiferous tubules were counted (**F**). The presence of VASA-, GFR-α-1-, α-6-Integrin-, CD9-, and C-KIT-positive stained cells in the seminiferous tubules of CT and CP-treated immature mice (**G**–**K**) was examined by immunofluorescence staining (IF) using specific primary antibodies and Cy3 or Alexa-flour 488 with the relevant secondary antibodies (VASA, α-6-Integrin, CD9, and C-KIT red staining and GFR-α-1 green staining). DAPI (blue color) stained the nucleus of the cells. Arrows show the location of stained cells in the testicular tissues. As a negative control (NC), we stained the tissues only with the secondary antibodies (NC for α-6-Integrin, CD9 and C-KIT were similar and therefore, we present only NC for α-6-Integrin). (**B**)—X20 light microscope magnification (100 µm scale). (**D**)—X40 light microscope magnification (100 µm scale). (**G**–**K**)—X40 fluorescent microscope magnification (100 µm scale). **—*p* < 0.01 and ***—*p* < 0.001.

Our results show that CP treatment of immature mice (CP) significantly decreased (*p* < 0.001) their testicular weight during 5 weeks after the last injection compared to control (CT) (Figure 1A). CP treatment also impaired seminiferous tubules histology during 3 weeks after the last injection (Figure 1B). The germinal epithelium was decreased (the cell layer decreased and the diameter of the lumen increased in tubules of CP-treated immature mice compared to CT), and seminiferous tubules appeared empty of most of the cells 1 and 3 weeks post treatment as compared to CT (Figure 1B). The most severe damage for seminiferous tubules can be seen 1–2 weeks post-treatment with CP compared to CT (Figure 1C,D). Afterwards, testis could restore, and after five weeks post-treatment, the histology of the STs were similar to control group (Figure 1B,C). Next, we examined the effect of CP at day 10 post-treatment on seminiferous histology (Figure 1D), testes weight (Figure 1E), and testes total cell count (Figure 1F). Our results show that CP treatment (CP) of immature mice significantly decreased the testicular weight 10 days after the last CP-treatment (Figure 1E; *p* < 0.001) and testicular cell count (Figure 1F; *p* < 0.001) compared to control (CT). In addition, the number of VASA, GFR-α-1-, α-6-Integrin, CD9, and C-KIT-positive stained cells/tubule (as a premeiotic cell marker; [2,46,49]) was significantly reduced in testicular tissue of CP-treated immature mice compared to CT (Figure 1).

### 2.2. CP-Treated Immature Mice Showed a Significant Decrease in the Number of Subpopulation of Spermatogenic Cells

**Figure 2:**
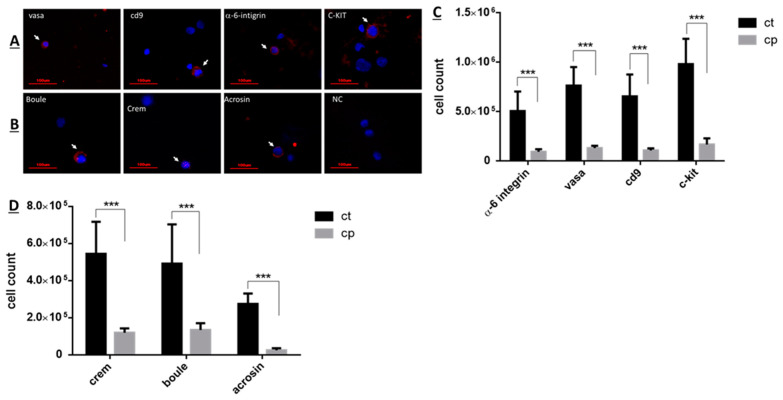
CP-treated immature mice showed a significant decrease in the number of subpopulations of spermatogenic cells compared to control: Cyclophosphamide (CP)- or PBS-treated mice (control, CT) were i.p injected as described in Figure 1. Ten days post-treatment, testes were removed, seminiferous tubules were separated, and cells were enzymatically isolated from the seminiferous tubules. The premeiotic cells that express α-6-INTEGRIN, VASA, CD9, GFR-α, and c-KIT, or the meiotic cells that express the markers BOULE and CREM and the meiotic/post-meiotic cells that express the marker ACROSIN were identified by immunofluorescence staining using specific primary antibodies for each cell marker and the secondary antibody Cy3 (red color). DAPI (blue color) stained the nucleus of the cells ((**A**,**B**), respectively). The identified premeiotic, meiotic, and meiotic/post-meiotic cells were counted, and their number/testis was evaluated ((**C**,**D**), respectively). Arrows indicate the stained cells. ***—*p* < 0.001.

The effect of CP on the number of subpopulation of spermatogenic cells (pre-meiotic and meiotic/post-meiotic cells) isolated from seminiferous tubules of immature mice was examined 10 days after the last CP-injection by using immunofluorescence staining specific for each type of cells premeiotic cells (Figure 2A) and meiotic/post-meiotic cells (Figure 2B) [2,46,49]. Our results show that CP treatment (cp) significantly reduced the number of premeiotic cells that specifically stained to α-6 INTEGRIN, VASA, CD9, and c-KIT compared to control (ct) (Figure 2C; *p* < 0.001). Moreover, CP treatment significantly reduced the number of meiotic cells that specifically stained to CREM and BOULE markers and to the meiotic/post-meiotic marker (ACROSIN) compared to control (Figure 2D; *p* < 0.001).

### 2.3. Effect of CP on the Number of Sertoli and Peritubular Cells and on the Functionality of Sertoli Cells from Immature Mice

**Figure 3:**
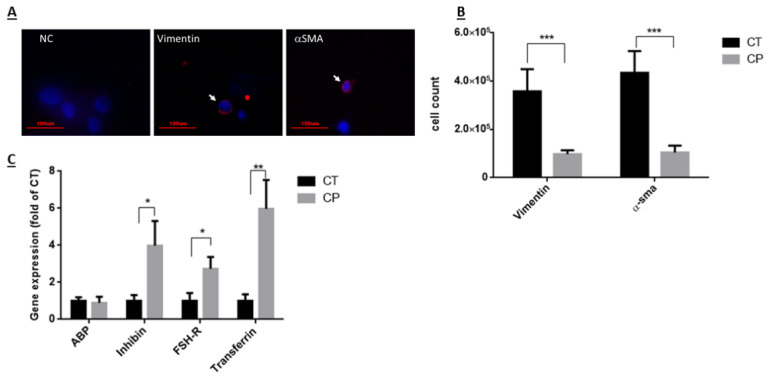
Effect of CP on the number of Sertoli and peritubular cells and on the functionality of Sertoli cells from immature mice: Cyclophosphamide (CP)- or PBS-treated mice (control, CT) were i.p injected as described in Figure 1. Ten days post- treatment, testes were removed, seminiferous tubules were separated, and cells were enzymatically isolated from the seminiferous tubules. Sertoli cells and peritubular cells were identified by immunofluorescence staining using specific antibodies for each cell type (vimentin—a marker for Sertoli cells and α-SMA—a marker for peritubular cells) and the secondary antibody Cy3 (red color) (**A**). DAPI (blue color) stained the nucleus of the cells (**A**). The identified cells were counted and their number/testis was evaluated (**B**). Moreover, RNA was extracted from cells isolated from seminiferous tubules of CT or CP-treated mice, and examined by qPCR analysis for the expression levels of factors known to be produced by Sertoli cells [androgen binding protein (ABP), inhibin, FSH-receptor (FSH-R), transferrin], using specific primers for each factor (**C**). Arrows indicate the stained cells. *—*p* < 0.05, **—*p* < 0.01, and ***—*p* < 0.001.

The effect of CP on the number of Sertoli and peritubular cells isolated from seminiferous tubules of immature mice was examined 10 days after last CP-injection, as examined by using specific immunofluorescence staining for each type of cells (VIMENTIN—a marker of Sertoli cells and αSMA—a marker of peritubular cells) (Figure 3A). Our results show that CP treatment (CP) significantly reduced the number of Sertoli and peritubular cells compared to control (CT) (Figure 3B; *p* < 0.001). On the other hand, evaluating the effect of CP on the functionality of Sertoli cells by examining the expression levels of some functional factors (known to affect spermatogenesis) produced by Sertoli cells, show that CP treatment significantly increased the expression levels of inhibin, FSH-receptor (FSH-R) and transferrin compared to control (Figure 3C; *p*< 0.05, 0.05, and 0.01, respectively), but did not significantly affect the expression levels of androgen binding protein (ABP) compared to control (Figure 3C).

### 2.4. Effect of Hormones (FSH and Testosterone) and Cytokines (IL-1α and TNFα) on the Proliferation and Differentiation of Spermatogonial Cells from CP-Treated Immature Mice In Vitro in MCS

**Figure 4:**
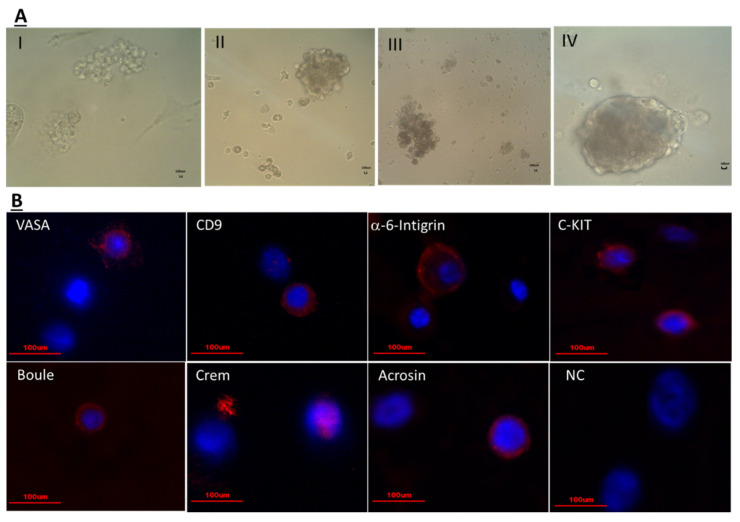
Isolated cells from seminiferous tubules of CP-treated immature mice developed colonies in vitro in methylcellulose culture system (MCS): Isolated cells from seminiferous tubules of CP-treated immature mice, ten days after the last injection were cultured in a methylcellulose culture system (MCS). The MCS was composed of 42% methylcellulose, KSR (10%), StemPro, and growth factors (GDNF, LIF, FGF, EGF) as described in materials and methods section in the absence or presence of IL-1α, TNF-α, FSH, testosterone (T), or both IL-1α + T, TNF-α + T, FSH + T. Developed colonies after 4–5 weeks of culture are presented (**A**). The developed cells in the different treatments were positively stained for premeiotic markers (VASA, CD9, α-6-integrin, C-KIT), meiotic markers (Boule, Crem) and meiotic/post-meiotic marker (Acrosin) as examined by immunofluorescence staining using specific primary antibodies for each cell type and the secondary antibody Cy3 (red color) and DAPI (blue color) that stained the nucleus of the cells (**B**). Scale bare: 100 μm.

Our results show that isolated cells from seminiferous tubules of immature mice ten (10) days post CP treatment still have spermatogenic cells that were positively stained for pre-meiotic (VASA, CD9, GFR-α, α-6-INTEFRIN, and c-KIT), meiotic (BOULE and CREM), and to meiotic/post-meiotic (ACROSIN) markers [before culture (BC)] as examined by immunofluorescence staining using specific antibodies for each marker (Figure 2A,B) and summarized in Table 1. In the present study, we were able to induce the proliferation (production of colonies; Figure 4A) and differentiation [a significant increase in the number of c-KIT and ACROSIN positive cells (after culture; AC) of the spermatogonial cells in vitro in MCS, compared to control (Table 1) (Figure 4B)]. The morphology of the developed colonies in the 3D culture was different, without relation to the type of treatment (I, II—colonies with medium size and different morphology, III—colonies with small size, IV—a big colony with attached marginal cells and detached cells). The cells in the developed cultures contained cells of the premeiotic (VASA, CD9, α-6-integrin, C-KIT), meiotic (Boule, Crem), and meiotic/post-meiotic stages (Acrosin). Our results show that in vitro culture of the isolated cells in MCS maintain the percentage of the premeiotic cells VASA, CD9, and GFR-a, but significantly decreased the percentage of a-6-INTEGRIN cells compared to before culture (BC) (Table 1). On the other hand, the percentage of the meiotic cells BOULE and CREM did not change compare to BC (Table 1). However, the percentage of the meiotic/post-meiotic ACROAIN-positive cells was significantly increased in MCS (AC) compared to BC (Table 1).

The addition of FSH in vitro did not show any significant effect on the percentage of premeiotic, meiotic, and meiotic/post-meiotic examined cells developed in MCS compared to AC (Table 1). However, the addition of testosterone (T) in vitro significantly increased the percentage of the premeiotic CD9 positive cells and the meiotic/post-meiotic ACROSIN positive cells, without any significant effect on the other examined premeiotic and meiotic cells developed in MCS compared to AC (Table 1). Addition of both FSH + T in vitro significantly decreased the percentage of the premeiotic CD9 positive cells compared to T, and the meiotic/post-meiotic ACROSIN positive cells developed in MCS compared to FSH or T (Table 1). However, the addition of FSH + T did not significantly affect the percentage of the other examined pre-meiotic and meiotic cells developed in MCS compared to FSH or T (Table 1).

Addition of IL-1α in vitro did not show any significant effect on the percentage of the examined premeiotic, meiotic, and meiotic/post-meiotic cells developed in MCS compared to AC (Table 1). However, addition of both IL-1α + T in vitro significantly increased the percentage of the premeiotic VASA positive cells and the meiotic BOULE positive cells developed in MCS compared to IL-1α or T, without any significant effect on the other examined premeiotic and meiotic/post-meiotic cells developed in MCS compared to T (Table 1).

The addition of TNFα in vitro significantly increased only the percentage of the premeiotic CD9 positive cells, without any significant effect on the other examined premeiotic and meiotic or meiotic/post-meiotic positive cells developed in MCS compared to AC (Table 1). However, the addition of both TNFα + T in vitro significantly decreased the percentage of the meiotic/post-meiotic ACROSIN positive cells without any significant effect on the other examined premeiotic or meiotic cells developed in MCS compared to T (Table 1).

## 3. Discussion

The harmful effect of chemotherapy/radiotherapy on prepubertal and pubertal male fertility is well recognized [49,50]. While fertility preservation of male pubertal patients is possible by sperm cryopreservation before chemotherapy/radiotherapy, the fertility preservation strategies for prepubertal cancer patients, who do not yet produce sperm, is still experimental [37,38,39,40,41,42,51]. In addition, most of the studies to evaluate the effect of chemotherapy/radiotherapy on the development of spermatogenesis and testicular cell functionality were performed in adult rodents [26,52,53,54,55]. On the other hand, the cellular components and functionality of the testis of immature and adult males are different, and thus the effect of chemotherapy/radiotherapy on these cells could be different.

In the present study we examined the in vivo effect of cyclophosphamide, an anti-cancer chemotherapy used in prepubertal patients, on the development of spermatogenesis in immature mice, and the possible use of the survivor spermatogonial cells to develop spermatogenesis in vitro using 3D MCS. Our results showed that CP treatment of immature mice led to a significant reduction in their testicular weight for 5 weeks post the last injection, and in the normal histology of the seminiferous tubules for 4 weeks post the last injection compared to control group. This could be related to the reduction in the number of spermatogonial, Sertoli, and peritubular cells as shown in Figure 1, Figure 2 and Figure 3 (these cells proliferate at the age when the CP was injected, therefore their numbers were reduced by the chemotherapy). Our results showed a significant reduction in the number of VASA, GFR-α-1, α-6-INTEGRIN, CD9, and C-KIT cells/tubule (as representative of testicular germ cells/spermatogonial cells) in CP-treated immature mice compared to control (Figure 1). A significant reduction was demonstrated in different subpopulations of testicular germ cells and in the meiotic and meiotic/post-meiotic cells (Figure 2). These results indicate that testicular germ cells of immature mice are very sensitive to the effect CP even though some of these cells survive the CP treatment. The reduction in meiotic and post-meiotic cells following CP treatment could be related to the reduction in the numbers of testicular dividing germ cells (premeiotic cells that mitotically divide) (some of the germ cells that already passed this stage of division continue their differentiation). It is possible that CP may directly affect the meiotic/post-meiotic cells and thus lead to additional decrease in their count. These results are in harmony with the study of Smart et al., 2018, that showed a significant reduction in germ cells in testicular fragments from immature mice that were cultured in vitro in the presence of CP, cisplatin, or doxorubicin [56]. Our results are in correlation with the study of Velez de la Calle et al., 1989, who showed a significant decrease in testicular weight and spermatogonial cells of rats (in different ages) treated with CP [57]. On the other hand, our results showed a significant reduction in the number of Sertoli cells and peritubular cells compared to control (Figure 3). This may indicate a direct effect of CP on the dividing Sertoli and peritubular cells. These cells involved in the construction of the seminiferous tubules and also considered as supporting cells (mainly Sertoli cells) in the process of spermatogenesis. Therefore, reduction in their number may affect the normal development of the seminiferous tubules and the process of spermatogenesis in the adult/puberty age. Our results are contradicting to the study of Smart et al., 2018 [56], who showed that addition of CP, cisplatin, or doxorubicin to testicular fragments of immature mice in vitro did not affect the number of Sertoli cells [56]. This contradiction could be related to the in vitro system this group used compare to our in vivo system. In this regard, our results should be considered during chemotherapy/radiotherapy treatment of prepubertal male patients in relation to their future fertility. In addition, we showed that CP affected not only the number of Sertoli cells, but also their functionality (Figure 3). CP significantly increased the expression of inhibin B, FSH-R, and transferrin, but did not affect the expression levels of ABP compared to control. These changes could be related to a direct effect of CP on Sertoli cells and/or as a result of reduction in germ cell counts and types, which were involved in regulation of Sertoli cell functions through cell-cell interaction. In addition, the changes in the expression levels of these factors may affect the cell-cell interactions in the seminiferous tubules and thus to impair the process of spermatogenesis, and thus lead to subfertility or infertility. Indeed, treatment of adult mice with busulfan that led to loss of spermatogonial cells significantly increased the expression levels of testicular GDNF [58]. Moreover, in vitro removal of bovine spermatogonial cells significantly increased the expression levels of GDNF and FGF2, but significantly decreased the expression levels of kit ligand [59]. Following germ cell depletion (mice treatment with busulfan) a significant increase was detected in the chemokine Cxcl12 (involve in the maintenance of spermatogonial cells) and a decrease in the expression of the receptor Cxcr7 [60]. Moreover, a depletion of germ cells leads to changes in the expression of some factors that produced by Sertoli cells as the following: 2/26 of the examined factors showed a decrease, 9/26 of the examined factors showed an increase, while 13/26 of the examined factors did not show any changes, and 2/26 of the examined factors showed biphasic response [16]. These results may indicate that germ cells play a key role in the regulation of Sertoli cell activity. The reduction in the numbers of these germ cells may affect the regulatory mechanisms of Sertoli cell activities, including changes in the levels of factors that involved in the regulation of germ cell development to generate complete spermatogenesis and thus to impair spermatogenesis development [17]. It is already known from the literature that SSC regulate Sertoli cells: Sertoli cells produce GDNF, which has a role in survival and self-renewal of germ cells and the expression of its receptor, GFR-α, by SSC serves as a key regulator for the process [61,62]. In addition, the SSC also regulate Sertoli cell’s function by activating the NOTCH signaling [62,63]. The NOTCH signaling components were found on both Sertoli and SSC, and this pathway may play a key role in the differentiation and survival of SSC [62,64]. The reduction in SSC impairs the NOTCH signaling pathway that eventually causes an upregulation of factors secreted by Sertoli cells. Furthermore, it was demonstrated that the NOTCH targets HES1 and HEY1, which are transcriptional repressors, directly downregulate GDNF expression [65]. Our results are in contradiction to previous study by Velez de la Calle et al., who could not show a significant effect of CP on Sertoli cells or Leydig cells functionality [57]. This could be related to using rats (not mice) and different protocol of CP treatment.

Fertility preservation for prepubertal males treated with gonadotoxic agents is still experimentally, and today there is no safe technology to offer to them. In vitro maturation studies using spermatogonial cells are still invalid, and most of these experiments were performed using spermatogonial cells from normal animals [2,8,39,62,66,67]. Recently, we published a proof of concept to induce spermatogenesis in vitro to generate sperm-like cell in 3D MCS from spermatogonial cells of busulfan-treated immature mice [46] and chemotherapy treated cancer patient boys [48]. In the present study, we were able to induce, for the first time, proliferation of premeiotic cells, and development of meiotic/post-meiotic cells (ACROSIN positive cells) in vitro from spermatogonial cells isolated from CP-treated immature mice [after culture (AC) compared to before culture (BC); Table 1]. These results may indicate that the survivor spermatogonial cells from CP-treated immature mice are biologically active and could proliferate and differentiate under in vitro conditions. On the other hand, only the addition of testosterone (T) to these cultures could significantly increase the percentages of CD9-positive cells (premeiotic cells) and ACROSIN-positive cells (meiotic/post-meiotic cells) in vitro compared to BC, while addition of FSH did not affect the percentages of premeiotic or meiotic and post-meiotic cells in these cultures (Table 1). Furthermore, addition of both FSH and T (FSH + T) to these cultures decreased the induction effect of T on CD9 and ACROSIN-positive cells (Table 1). These results may suggest a stimulatory role for T in the development of spermatogenesis in vitro, while FSH antagonize this effect. The mechanism/s behind this feature need to be explored in future studies. Moreover, the addition of testicular paracrine/autocrine factors (IL-1α or TNF-α), that also produce by testicular cells under physiological conditions, to these cultures showed no effect of IL-1α on the percentages of premeiotic or meiotic/post-meiotic cells, while TNF-α significantly increased the percentage of CD9-positive cells compared to BC (Table 1). IL-1α is known to have key role in paracrine/autocrine regulation [10,11,12] and showed induction of DNA synthesis and differentiation of spermatogonial cells in rats [14,15]. TNF-α known to induce SSCs survival [68] and production of blood–testes barrier [11,29]. The addition of IL-1α and T (IL-1α + T) significantly increased the percentages of VASA- (premeiotic cells) and BOULE-positive cells (meiotic cells) compared to IL-1α or T, while the addition of TNF-α and T (TNF-α + T) lead to a significant decrease in the percentage of acrosine-positive cells without any effect on the other examined cells (Table 1). These results may suggest an additive/synergistic effect of IL-1α and T in the development of spermatogenesis in vitro, while TNF-α may reduce the stimulatory effect of T in this process. Our results may indicate the different effect/involvement of IL-1α and TNF-α in the development of spermatogenesis in vitro. The mechanism/s behind these effects in vitro and/or in vivo should be examined in future studies.

The developed colonies from normal immature mice were shown to contain Sertoli cells, peritubular cells, and premeiotic, meiotic, and post-meiotic cells as examined by confocal microscopy using specific markers for each cell type (tubular-like structures) (submitted for publication). In addition, we showed the development of 1N, 2N, and 4N cells in the 3D cultures as examined by FACS (submitted for publication). Sertoli cells are present in the 3D culture and therefore, addition of testosterone or FSH can be involved in the development of different stages of spermatogenesis in vitro. Under some conditions, we were able to induce the development of premeiotic cells, but not meiotic or post-meiotic cells. This could be related to induction of in vitro conditions that induce proliferation, but not differentiation of the premeiotic cells.

Thus, we were able to induce the spermatogonial cells from CP-treated immature mice to proliferate and differentiate to meiotic and post-meiotic stages, but not to generate sperm-like cells under in vitro culture conditions of MCS. Testosterone was the most effective inducer of spermatogenesis in vitro, while combination of T + IL-1α increases this effect. However, combination of T + TNF-α or T + FSH decreased this effect. These results clearly show the involvement of endocrine and paracrine factors in the regulation the development of spermatogenesis in vitro. It is possible to suggest that optimization of the in vitro system should include a balance between these and other factors involved in normal spermatogenesis in vivo.

In summary, our results show that CP treatment of immature mice significantly decrease the numbers of testicular germ cells and the somatic Sertoli and peritubular cells. In addition, it affects the functionality of Sertoli cells, and thus may affect the microenvironment surrounding the testicular germ cells. This may lead to impairment in the survival, proliferation and differentiation of spermatogonial cells, and thus to subfertility or infertility. Our present and previous studies using human, monkey, and mouse spermatogonial cells may suggest, at least in principle, that it is possible to induce the proliferation and differentiation of these cells from normal and pathological conditions, including chemotherapy, to meiotic and post-meiotic stages, and in some cases to develop sperm-like cells using 3D in vitro culture systems [2,39,44,45,46,47,48,68]. Successful optimization of this in vitro 3D system may provide possible tool/technology to develop functional sperm and thus, to be used for fertility preservation of prepubertal male patients who schedule for aggressive chemotherapy/radiotherapy treatment, and also for some cases of infertile nonobstructive azoospermic men. On the other hand, the genetic and epigenetic of the developed cells and the generated sperm should be carefully examined and confirmed before clinical translation.

## 4. Materials and Methods

### 4.1. Animals

The present study was confirmed by the Ben-Gurion University Ethics Committee for Animal Use in Research (IL-17-11-2014). Sexually immature 7-day-old ICR male mice were purchased from Envigo Laboratories, Jerusalem, Israel. Experiments were performed two to three days after arrival of the mice.

### 4.2. Cyclophosphamide Administration

Cyclophosphamide (CP) powder (MP Biomedicals, Illkirch, France) was dissolved in sterile PBS. 100 µL of CP (100 mg/kg) (CP group; *n* = 150 mice) or 100 µL PBS (control group; CT; *n* = 75 mice) were intraperitoneally (i.p) injected into the immature mice. Injections were performed once a week for 3 weeks. One week to 5 weeks after the last injection (post-treatment), mice were sacrificed by CO_2_. This protocol was performed according to Carmely et al., 2009 [69]. The dose of CP that was used is in harmony to the therapeutic dose of humans [32]. Testes were weighed and fixed in Bouins’ solution for histological evaluation. Ten days post the last injection (10 days post CP injection), mice were sacrificed and testes were fixed for histological evaluation and/or frozen in −70 °C for RNA extraction or were immediately used for tubular cell isolation and for cellular evaluations and for in vitro culture in MCS.

### 4.3. Tubular Cell Isolation

The procedure was performed under sterile conditions. Testes from both control and CP-treated mice were removed, and tunica albuginea was gently removed by scalpel knife. Seminiferous tubules (STs) were immersed with PBS and thereafter transported through a syringe 2–3 times to complete the mechanical digestion. Thereafter, the STs were enzymatically digested as described by AbuMadighem et al., 2018 [46].

### 4.4. In Vitro Culture of Tubular Cells

Cells were isolated from 20 CP-treated mice for experiment. Cells were seeded in plates of 24 wells. Each well contained 200,000 cells/500 µl suspended in media-containing StemPro (33%) (Gibco, Waltham, MA, USA), KnockOut serum replacement-KSR (25%) (Gibco, USA), growth factors such as human rEGF (recombinant epidermal growth factor) (20 ng/mL) (Biolegend, San Diego, CA, USA), human rGDNF (glial cell line derived nerve growth factor) (10 ng/mL) (Biolegend), human rLIF (leukemia inhibitory factor) (10 ng/mL) (Biolegend), and human r-bFGF (basic fibroblast growth factor) (10 ng/mL) (Biolegend), penicillin-streptomycin (pen-strep, 1%), and methylcellulose (MC; 42%) (R&D, McKinley Place NE, Minneapolis, MN, USA) as described by AbuMadighem et al., 2018 [46]. From the beginning of the cultures we added follicle stimulating hormone (FSH) (7.5 IU/mL) (Serono, Geneva, Switzerland) or interleukin 1 alpha (IL-1α) (20 pg/mL) (BioLegend) or tumor necrosis factor alpha (TNF-α) (20 pg/mL) (BioLegend). However, testosterone (T) (10^−7^ M) (Bayer, Hod Hasharon, Israel) alone or in combination with other factors (FSH + T, IL-1α + T or TNFα + T) was added at the last week of the culture. The above-mentioned growth factors were just added every 7–10 days in a ×10 concentration (50 μL/well) to the cultures. The cells were usually cultured in MCS for 4–5 weeks.

### 4.5. Histological and Immunostaining of Testicular Tissues and Cells

Slide preparation and fixation of testicular tissues and cells, immunostaining, and hematoxylin-eosin tissue staining were performed as described by AbuMadighem et al., 2018 [46].

Following the removal of the blocking buffer, the first antibodies were added, as follows: Monoclonal mouse anti-mouse Vimentin (Novus, Littleton, CO, USA; 1:500), and polyclonal goat anti-mouse α- sma (Abcam, 1:250), Polyclonal goat anti-mouse Integrin α6 (Santa Cruz, CA, USA; 1:40), polyclonal rabbit anti-mouse VASA (Santa Cruz; 1:100), polyclonal rabbit anti-mouse CD9 (Santa Cruz; 1:100), monoclonal mouse anti-mouse GFR-α-1 (Santa Cruz, sc-271546; 1:50), monoclonal mouse anti-mouse α-6-INTEGRIN (Santa Cruz, 1:50), monoclonal mouse anti-mouse CD9 (Santa Cruz, 1:50), and monoclonal mouse anti-mouse C-KIT (Santa Cruz, 1:50), polyclonal rabbit anti-mouse BOULE (Santa Cruz; 1:50), polyclonal rabbit anti-mouse CREM-1 (Santa Cruz; 1:50), and polyclonal rabbit anti-mouse ACROSIN (Santa Cruz; 1:200). Following overnight incubation at 4 °C, the slides were washed, and the specific secondary antibodies were added compatibly to the first antibodies (goat anti-mouse IgG (Rhodamine red), donkey anti-goat IgG (Cy3), Alexa-flour 488; Jackson Immuno Research (West Grove, PA, USA)) for 40 min at room temperature. After washing, the slides were dried and DAPI, which stains the nuclei blue, was added to the tissues, and the cover slides were applied. The negative control was incubated in a blocking buffer instead of the first antibody.

### 4.6. Cell Count of the Stained Cells/Testis

Cells were enzymatically isolated from the seminiferous tubules of CT and CP-treated mice. The number of cells/testis was determined. Thereafter, the cells were fixed and stained for the different types of cell markers, and the percentage of the positive cells for each marker was determined. The count of specific cell type/testis was determined by multiplying the percentage of the specific cell type by the total cells isolated from the testis.

### 4.7. Microscope Analysis

Preformed by Olympus IX70 microscope (Olympus, Novato, CA, USA). Digital images were prepared using Image-Pro Plus (Media Cybernetics, Bethesda, MD, USA), Microsoft Excel, and Adobe Photoshop 7.0 software.

### 4.8. Gene Expression

RNA was extracted from isolated testicular cells, from control or CP-treated immature mice, by GenElute Mannalian Total RNA Miniprep Kit (Sigma, St. Louis, MO, USA).

cDNA synthesis was performed according to the qScript cDNA Synthesis Kit (Quantabio, Beverly, MA, USA), and qPCR was performed using specific primers for each examined marker: ABP (forward: GCAGCATGAGGATTGCACTA; reverse: CATGAGGCTGGGGAATGTCT; product size, 237 bp), INHIBIN B (forward: CCTGTCATCAGGGCAAGTGA; reverse: TCGAGGCAGACGCCTTATTC; product size, 209 bp), FSH-R (forward: GTGCATTCAACGGAACCCAG; reverse: AGGGAGCTTTTTCAAGCGGT; product size, 206 bp), TRANSFERRIN (forward: CCAAGCTCCAAACCATGTTGT; reverse: ACAGATTGCATGTACTCCGCT; product size: 231 bp), Housekeeping gene GAPDH (forward: ACCACAGTCCATGCCATCAC; reverse: CACCACCCTGTTGCTGTAGCC; product size, 450 bp). qPCR reaction was performed following the 2x qPCRBIO SyGreen Blue Mix Hi-ROX (PCR Biosystems Ltd., Aztec House, 397-405 Archway Road, London, UK) protocol and was performed using the LightCycler 96 real-time PCR machine (Roche, Roche Diagnostics Corporation, Roche CustomBiotech, Indianapolis, IN, USA). Program: Preincubation 10 min at 95 °C, 40 cycles of 15 s at 95 °C, 15 s at 60 °C, and 10 s at 72 °C. Melting cycle: 10 s at 95 °C, 60 s at 65 °C, and 1 s at 97 °C. PCR products were identified by the melting curve. The “threshold cycle” (Ct) value for each transcript was undefined. The relative quantity of gene expression was analyzed by the 2^−ΔΔCt^ method. Results were expressed as the fold of increase related to the GAPDH of the same examined sample and relatively compared to control treatment group.

## Figures and Tables

**Table 1:** Effect of hormones (FSH and testosterone) and cytokines (IL-1α and TNFα) on the proliferation and differentiation of spermatogonial cells isolated from CP-treated immature mice cultured in vitro in MCS.

	Spermatogenic Markers							
	Pre-Meiotic					Meiotic/Post-Meiotic		
Treatment	VASA	CD9	GFR-a	a-6-INTG	C-KIT	BOULE	CREM	ACROSIN
BC	23.65 ± 5.79	14.88 ± 4.86	13.1 ± 2.33	28.05 ± 7.93	6.65 ± 1.25	31.25 ± 9.5	20.8 ± 6.2	10.3 ± 1.65
AC AC+	26.23 ± 4.26	19.56 ± 1.44	21.77 ± 1.9	14.86 ± 5.88 *	19.5 ± 1.68 *	19.16 ± 4.29	25.46 ± 7.51	35.96 ± 5.9 ***
FSH	24.04 ± 4.42	25.62 ± 2.38	18.97 ± 5.7	18.8 ± 5.47	22.42 ± 5.39	20.6 ± 4.09	35.16 ± 6.46	43.92 ± 5.65
T	23.76 ± 3.56	42.63 ± 5.19 ###	24.03 ± 4.22	12.23 ± 0.76	11.13 ± 2.09	22.15 ± 2.29	38.42 ± 6.47	55.53 ±6.86 ##
FSH + T	21.15 ± 2.02	21.02 ± 4.56 $$$	20.77 ± 4.36	20.87 ± 3.17	16.9 ± 2.91	27.03 ± 8.39	34.45 ± 6.2	25.67 ± 6.01 $$$, @@
IL-1	20.8 ± 5.33	23.4 ± 6.14	22.77 ± 4.23	17.5 ± 7.44	16.5 ± 4.05	27.16 ± 6.64	30.45 ± 8.12	34.27 ± 10.77
IL-1 + T	49.2 ± 8.3 $,@	48.36 ± 6.43	25.2 ± 6.57	17.93 ± 3.16	20.1 ±5.83	61.12 ± 5.1 $$$, @@@	42.82 ± 5.12	55.09 ± 7.61
TNF	31.67 ± 6.16	33.5 ± 4.29 #	25.3 ± 4.1	25.76 ± 4.61	29.05 ±5.51	21.1 ± 4.01	36.1 ±9.1	36.4 ± 7.32
TNF + T	28.92 ± 11.24	43.63 ± 11.03	27.2 ± 10.9	34.9	27.7 ± 4.4	16.76 ± 6.16	35.1 ± 16.58	34.52 ± 10.79 $$

Ten days after the last injection of CP (see Figure 1 and Figure 2), testes were removed, seminiferous tubules were separated, and cells were enzymatically isolated and cultured in MCS as described in Figure 4. In some wells, we also added from the beginning of the culture IL1-α (20 pg/mL), TNF-α (20 pg/mL), FSH (7.5 IU/mL). However, testosterone (T; 10^−7^M) was added only at the last week of the culture, alone or in combination as the following: IL-1α + T, TNF-α + T, FSH + T. Every 10–14 days, we added new media containing the same composition of factors that was added in the beginning of the culture. After 4–5 weeks, the developed colonies and cells were collected, and the cells were fixed by cold methanol and stained by immunofluorescence staining using specific antibodies for markers of the premeiotic (VASA, CD9, GFR-α, α-6-INTEGRIN, c-KIT), meiotic (CREM, BOULE) and meiotic/post-meiotic (ACROSIN) cells. The percentage of cells stained (relative to all cells present in the counted field) for each examined marker in each treatment was evaluated. We compared the percentages of cells stained for the each examined marker after culture (AC) and before culture (BC). We also compared the effect of the different treatments (FSH, T, IL-1α, TNF-α, FSH + T, IL-1α + T, TNF-α + T) after culture (AC+) on the percentages of cells after culture (AC) from the different treatments. *—Compared to BC (*—*p* < 0.05; ***—*p* < 0.001), #—Compared to AC (#—*p* < 0.05; ##—*p* < 0.01; ###—*p* < 0.001), $—Compared to T ($—*p* < 0.05; $$—*p* < 0.01; $$$—*p* < 0.001), @—Compared to FSH or IL-1α or TNF-α (according to the pair). (@—*p* < 0.05; @@—*p* < 0.01; @@@—*p* < 0.001). Green color—significant increase. Red color—significant decrease.

## Data Availability

The data that support the findings of this study are available from the corresponding author upon reasonable request.
